# Oxaliplatin-Induced DHX9 Phosphorylation Promotes Oncogenic Circular RNA CCDC66 Expression and Development of Chemoresistance

**DOI:** 10.3390/cancers12030697

**Published:** 2020-03-16

**Authors:** Ya-Chi Lin, Ya-Shan Yu, Hui-Hsuan Lin, Kuei-Yang Hsiao

**Affiliations:** 1 Department of Plant Pathology, College of Agriculture and Natural Resources, National Chung Hsing University, Taichung 40227, Taiwan; yachi.lin@gmail.com; 2 Department of Biotechnology, Asia University, Taichung 41354, Taiwan; 3 Institute of Biochemistry, College of Life Sciences, National Chung Hsing University, Taichung 40227, Taiwan; yashan.yu15@gmail.com (Y.-S.Y.); lhuihsuan@gmail.com (H.-H.L.); 4 Ph.D. Program in Translational Medicine, College of Life Sciences, National Chung Hsing University, Taichung 40227, Taiwan; 5 Rong Hsing Research Center for Translational Medicine, College of Life Sciences, National Chung Hsing University, Taichung 40227, Taiwan; 6 Bachelor Program of Biotechnology, College of Agriculture and Natural Resources, National Chung Hsing University, Taichung 40227, Taiwan

**Keywords:** circular RNA, circCCDC66, DHX9 phosphorylation, chemoresistance, DNA damage response, oxaliplatin, colorectal cancer, PI3KK.

## Abstract

Circular RNA (circRNA), generated through backsplicing in which the downstream splice donor joins the upstream splice acceptor, is a novel class of RNA molecules. Our previous study found that a novel oncogenic circRNA—consisting exon 8–10 of *CCDC66*—is aberrantly expressed in colorectal cancer (CRC) tissues and cells. The failure of treatment for colorectal cancer is typically associated with recurrent and chemoresistant cancerous tissues. In this study, we aimed to investigate the role(s) of circCCDC66 during the development of chemoresistance. We discovered that the expression level of circCCDC66 is elevated in colorectal cancer cells with resistance to oxaliplatin. Knockdown of circCCDC66 caused the downregulation of a subset of genes which are regulated by circCCDC66-associated miRNAs and related to the modulation of apoptosis and the cell cycle, suppressing cell survival, promoting oxaliplatin-induced apoptosis and, thus, hindering the development of oxaliplatin-resistance (OxR). The induction of circCCDC66 was dependent on the time-course and dose of oxaliplatin treatment. Our analyses revealed that DHX9 harbors two phosphorylation sites of phosphatidylinositol 3-kinase-related kinases (PI3KKs) close to substrate-binding domains. Blockage of phosphorylation by either PI3KK inhibitors or nonphosphorable mutants of DHX9 decreased the oxaliplatin-induced circCCDC66 expression and the ability to develop chemoresistant cells. Taken together, we demonstrated and linked the functional role of DHX9 phosphorylation to oncogenic circCCDC66 expression during the development of resistance to oxaliplatin, providing a mechanistic insight for the development of therapeutic strategies to recurring/chemoresistant colorectal cancer.

## 1. Introduction

Colorectal cancer was the third-most common cancer globally with approximately 1.8 million new cases in 2018 [1], ranked as the second-leading cause of cancer-related death in the United States of America [2] and third in Taiwan [3]. The intervention for colorectal cancer patients includes surgical resection, irradiation and chemotherapy. Typically, chemotherapy is applied to patients with metastatic tumors or as adjuvant treatment after irradiation or surgical resection. However, the relapse of tumor tissues at remote locations with resistance to chemotherapeutic agents would ultimately cause the failure of treatment for colorectal cancer (CRC). Thus, it is urgent to discover new mechanisms underlying the development of chemoresistance and to identify novel therapeutic targets.

Circular RNAs, RNAs with a circular configuration, are a novel widespread class of regulatory RNA molecules with various biological activities and are produced by backsplicing, in which the splice acceptor of an upstream exon is linked to the splice donor of a downstream exon by canonical spliceosome machinery [4,5,6]. One of the most popularly studied functions of circular RNA (circRNA) is to serve as molecular sponge for miRNA/RNA-binding proteins to regulate the gene expression network [7,8,9]. It has been widely accepted that some of circRNAs modulate gene expression through the interaction with single or multiple miRNAs to regulate multiple biological pathways [8,10,11,12]. Oncogenic circCCDC66 was aberrantly expressed in the tumorous tissues of colorectum and exerted its oncogenic effect through the protection of multiple oncogenes from attacks of a set of miRNAs [12,13]. However, the underlying mechanism for the regulation of circCCDC66 expression and the potential therapeutic application in chemoresistant CRC remain to be determined.

The regulation of circRNAs highly depends on both the flanking introns and factors binding to these introns [14,15,16,17,18,19]. Among these factors, both Muscleblind (MBL) and Quaking (QKI) proteins recognize particular motifs within the flanking introns and facilitate the interaction between introns. In addition to the model of recognition of specific motifs by MBL or QKI, DExH-Box Helicase 9 (DHX9) harboring ATP-dependent RNA helicase activity was reported to unwind the pairing between upstream and downstream complementary sequences and, thus, inhibited circRNA biogenesis [17]. Intriguingly, DHX9 is essential for tumorigenesis [20,21,22], but how it may promote or suppress the oncogenic circRNAs requires further characterization.

One of the molecular mechanisms of how oxaliplatin kills cells is to induce DNA damage through multiple routes [23,24]. To cope with various types of genotoxic stress, multiple pathways are activated to coordinate cell cycle arrest/resumption, global transcription/translation shutdown and DNA repair through the activation of PI3KKs [25,26,27,28]. Interestingly, it was reported that DHX9 is phosphorylated by and associated with one of the PI3KKs—DNA-dependent protein kinase upon DNA damage [29]. A parallel study also found that DHX9 is a substrate for ATM/ATR [30], the other two members of the PI3KK family. These studies suggest that the functions of DHX9 may be regulated by PI3KKs-mediated phosphorylation during the DNA damage response. However, the role of DNA damage-modulated DHX9 in the regulation of circRNA expression during chemoresistance remains unknown.

In this study, we first identified the novel molecular mechanism that the elevated level of circCCDC66 in chemoresistant CRC cells was regulated by the oxaliplatin-induced DHX9 phosphorylation. It provided an insightful mechanistic view how genotoxic stress-impaired DHX9 may help tumor cells escape from genotoxic stress through promoting oncogenic circRNA expression.

## 2. Results

### 2.1. The Expression Level of circCCDC66 Is Elevated in the Oxaliplatin-Resistant Colorectal Cancer Cells

**Figure 1:**
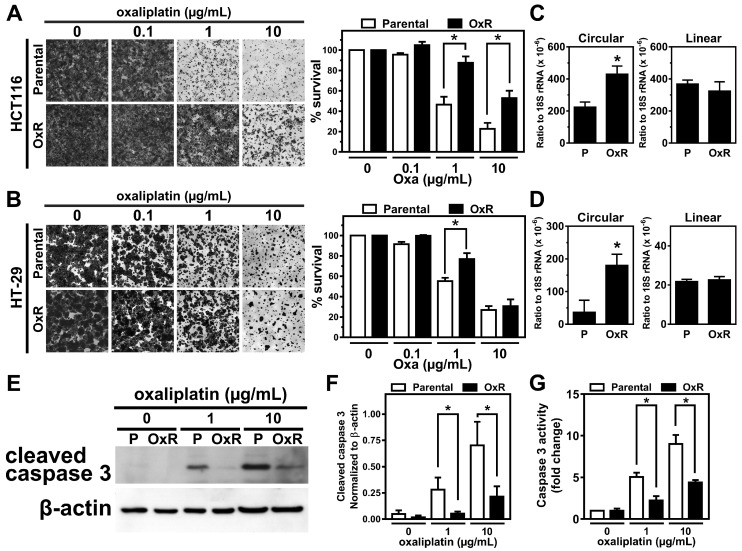
The level of circCCDC66 is elevated in the oxaliplatin-resistant cells. (**A**) The results of Coomassie blue staining from HCT116 cells without (parental cells) or with oxaliplatin resistance (OxR) treated with the indicated concentrations of oxaliplatin for 48 h. The right panel shows the quantitative results. (**B**) Similar to (A), performed in HT-29. (**C,D**) The levels of circular transcript and mRNA (linear transcript) of CCDC66 assayed by using RT-qPCR in HCT116 and HT-29 cells without/with oxaliplatin resistance. (**E**) The representative images for cleaved caspase 3 and β-actin from parental and oxaliplatin-resistant cells treated with the indicated concentrations of oxaliplatin for 48 h. (**F**) Quantitative results for (E). (**G**) Caspase 3 activities from cells with indicated treatments. * *p* < 0.05.

Our previous study has revealed that the colorectal tumor tissues expressed higher levels of circCCDC66 and preferentially regulated a subset of oncogenes in tumor transcriptome through the interaction with multiple miRNAs [12,13]. The progression and development of colorectal cancer typically become much more unmanageable after chemotherapy due to the development of chemoresistance. To address the potential roles of circCCDC66 in chemoresistant CRC, we first established the oxaliplatin-resistant (OxR) cell lines using both HCT116 and HT-29. The results from both HCT116 and HT-29 cells treated with a serial dosage of oxaliplatin demonstrated that there were more cells remaining for the OxR cells compared to the parental ones after the oxaliplatin treatment at the doses of 1 and 10 µg/mL (Figure 1A,B). To further investigate whether the expression of circCCDC66 is involved in the development of oxaliplatin resistance, we used the reverse transcription-quantitative PCR (RT-qPCR) and circCCDC66-specific primers to show that OxR cells have elevated levels of circCCDC66 in both HCT116 and HT-29 (Figure 1C,D, left), while no difference for mRNA of CCDC66 (linear transcript) between parental and OxR lines (Figure 1C,D, right). The resistant cells had lower levels of the cleaved form of caspase 3 and corresponding activities (Figure 1E–G), implying that circCCDC66 may play certain roles for cell survival during oxaliplatin-induced cell death.

### 2.2. The Elevated circCCDC66 Is Required for Cell Survival Against Oxaliplatin-Induced Apoptosis

**Figure 2:**
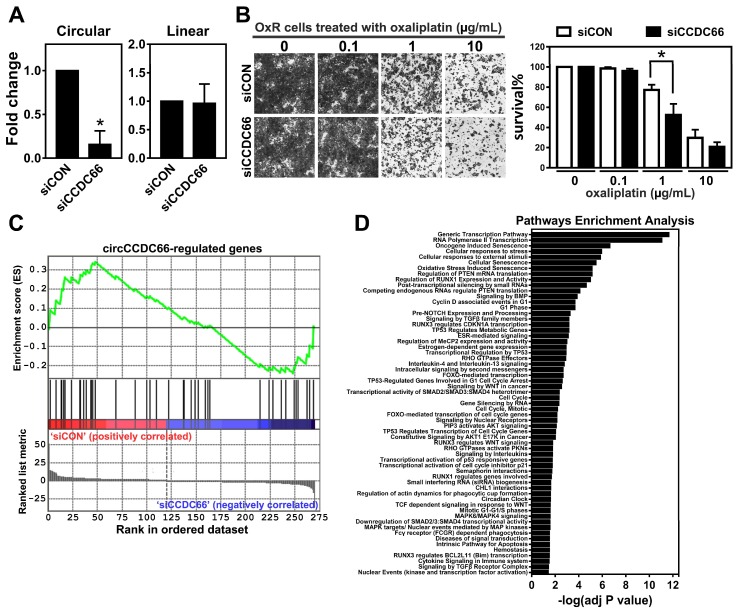
The expression of circCCDC66 is required for cell survival under oxaliplatin-induced genotoxic stress. (**A**) The levels of the circular transcript and mRNA (linear transcript) of CCDC66 assessed by qPCR in HCT116 transfected with control siRNA (siCON) or siRNA against circCCDC66 (siCCDC66). (**B**) The results of Coomassie blue staining from oxaliplatin-resistant (OxR) HCT116 transfected with control siRNA (siCON) or siRNA against circCCDC66 (siCCDC66) followed by a treatment with oxaliplatin at indicated concentrations for 48 h. Right panel: Quantitative results from the Coomassie blue staining. (**C**) Result of Gene Set Enrichment Analysis using the gene list ranked by fold change (siCCDC66/siCON) and circCCDC66 target genes. (**D**) Result of a pathway enrichment analysis using circCCDC66 target genes. * *p* < 0.05.

The oncogenic circCCDC66 modulates multiple genes promoting cell proliferation and survival [13]. To know whether the elevation of circCCDC66 expression is required for the survival advantage of oxaliplatin-resistant cells, the resistant cells treated with siRNA oligonucleotides targeting circCCDC66 or control oligonucleotides were subjected to the oxaliplatin treatment at doses of 0.1, 1 and 10 µg/mL. The qPCR results from cells treated with siRNAs showed the specificity and efficiency on the circular transcript but not the mRNA of CCDC66 (Figure 2A). The number of survival cells was significantly decreased in cells with circCCDC66 knockdown compared to cells with control oligonucleotides (Figure 2B). Furthermore, the genome-wide analysis using RNA sequencing on HCT116 OxR cells transfected with siRNA oligonucleotides targeting circCCDC66 or with control oligonucleotides revealed that previously predicted circCCDC66 target genes were enriched in differentially expressing genes mediated by circCCDC66 knockdown (Figure 2C). These genes were largely involved in multiple pathways related to the modulation of the responses to cellular stress, cell cycle progression and apoptosis (Figure 2D), supporting the notion that circCCDC66 controls the expression of multiple genes favoring cell survival through interactions with a set of miRNAs.

### 2.3. The Expression of circCCDC66 Is Induced by Treatment with Oxaliplatin

**Figure 3:**
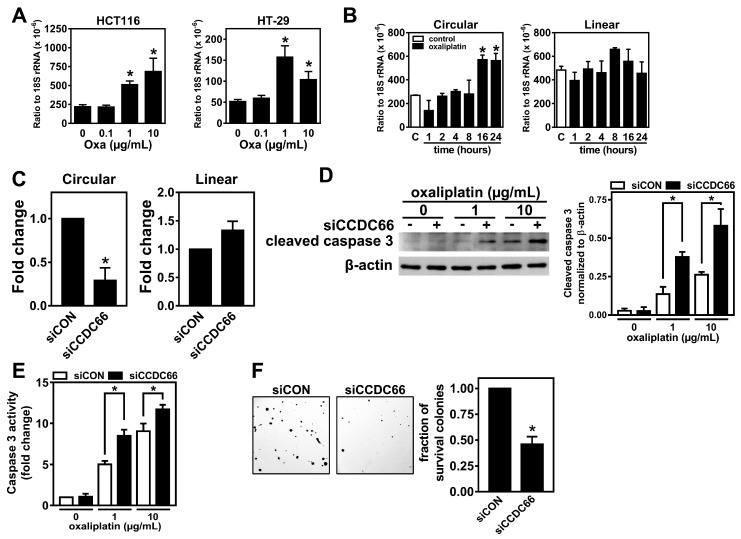
Expression of circCCDC66 is induced by oxaliplatin treatment. (**A**) Levels of circCCDC66 in CRC cell lines treated with oxaliplatin (Oxa) at indicated doses (Left: HCT116; Right: HT-29). (**B**) Levels of the circular transcript and mRNA (linear transcript) of CCDC66 in HCT116 treated with oxaliplatin (1 μg/mL) for the indicated times. (**C**) Levels of the circular transcript and mRNA (linear transcript) of CCDC66 from HCT116 transfected with control siRNA (siCON) or siRNA targeting circCCDC66 (siCCDC66). (**D**) The representative images for cleaved caspase 3 and β-actin from HCT116 cells transfected with control siRNA (siCON) or siRNA targeting circCCDC66 (siCCDC66) and followed by a treatment with oxaliplatin at the indicated doses for 48 h (left). Quantitative result is shown on the right panel. (**E**) Caspase 3 activities from cells with the indicated treatments. (**F**) Representative images for a clonogenic assay performed in HCT116 cells transfected with control siRNA (siCON) or siRNA against circCCDC66 (siCCDC66) and treated with 1-µg/mL oxaliplatin for more than 7 days. * denotes *p* < 0.05.

To characterize whether the elevated level of circCCDC66 in the OxR cells is directly induced by oxaliplatin treatment rather than a result of the selection of a circCCDC66-expressing population, we treated HCT116 and HT-29 cells with various doses of oxaliplatin, and the results demonstrated a dose-dependent induction of circCCDC66 in HCT116 (Figure 3A, left panel). Similarly, the oxaliplatin treatment also significantly induced the expression of circCCDC66 at doses of 1 and 10 μg/mL in HT-29 (Figure 3A, right panel). This induction of circCCDC66 (Figure 3B, left) but not the mRNA of the CCDC66 transcript (Figure 3B, right) depended on the time of the treatment with oxaliplatin, suggesting that the induction of circCCDC66 may be mediated through a post-transcriptional mechanism such as enhanced backsplice efficiency rather than transcriptional activation. In addition, the suppression of oxaliplatin-induced circCCDC66 expression using siRNA significantly increased oxaliplatin-induced cleaved caspase 3 and corresponding activities (Figure 3C–E), suggesting that the expression of circCCDC66 is required for cell survival under oxaliplatin-induced cellular stress. Furthermore, the knockdown of circCCDC66 decreased the colony formation in HCT116 (Figure 3F), suggesting that the induction of circCCDC66 is required for cell survival during the treatment with oxaliplatin and crucial for the establishment of a resistant population.

### 2.4. Oxaliplatin Promotes circCCDC66 Expression through DHX9 Phosphorylation

**Figure 4:**
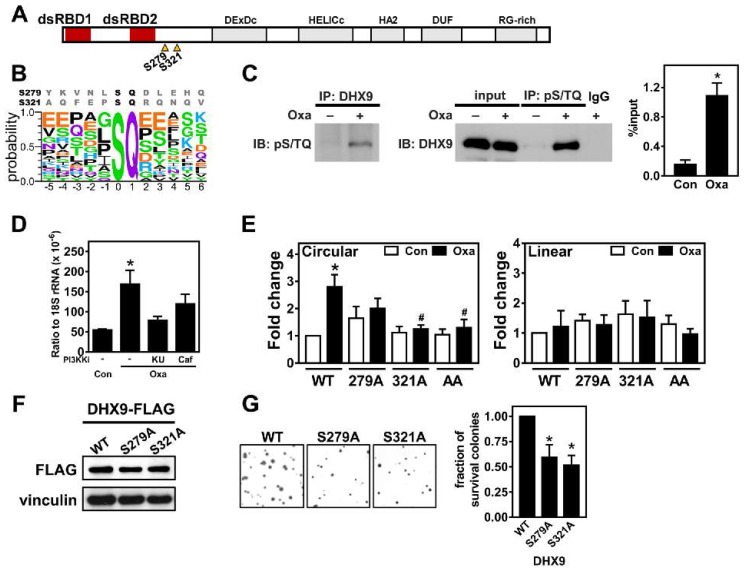
Oxaliplatin-induced DHX9 phosphorylation promotes circRNA expression. (**A**) Illustration of DHX9 domains. Triangles: Potential phosphorylation sites at serine 279 and 321. (**B**) Sequences around predicted PI3KK phosphorylation sites in DHX9 (top) compared to known sequences (Bottom image was generated by using WebLogo [31]. Y-axis: probability for each amino acid at the corresponding position and X-axis: position relative to phosphorylated serine). (**C**) Representative images of an immunoblot (IB) using an antibody recognizing the pS/TQ motif from immunoprecipitated (IP)-DHX9 (left). In a reciprocal fashion, representative images of a DHX9 immunoblot from immunoprecipitated proteins containing the pS/TQ motif (middle). Levels of phospho-DHX9 are shown as % of input (right). Input: lysates without IP. Oxa: cells treated without (–) or with (+) oxaliplatin (1 μg/mL) for 8 h. IgG: IP using rabbit nonimmune immunoglobulin. (**D**) Levels of circCCRC66 in the cells treated with indicated compounds for 24 h. Oxa: oxaliplatin (1 μg/mL), KU: KU-55933 (10 μM) and Caf: caffeine (1 mM). * *p* < 0.05, significantly different to control group. (**E**) The levels of the circular transcript and mRNA (linear transcript) of CCDC66 determined by quantitative PCR in oxaliplatin-treated HCT116 cells transfected with denoted DHX9 constructs (WT: wildtype; nonphosphorable versions: serine residue at 279 or 321 mutated to alanine or combination of both (AA)). * *p* < 0.05, significantly different to WT/con. **#** *p* < 0.05, significantly different to WT/oxa. (**F**) The representative images of immunoblots for HCT116 transfected with the DHX9 expressing vector. Vinculin serves as the loading control. (**G**) Similar to (E), representative images for a clonogenic assay performed in cells transfected with indicated DHX9 constructs and treated with 1-µg/mL oxaliplatin for more than 7 days. * *p* < 0.05, significantly different to WT.

One of the important regulators of circRNA biogenesis, DHX9, controls the pairing of intronic sequences flanking the circularizable region [17]. Our analyses found that there were two patches of sequences containing serine residue similar to the PI3KK substrate near the double-strand RNA binding domains (dsRBD1 and dsRBD2) of DHX9 (Figure 4A,B). To characterize whether these two potential phosphorylation sites may control the DHX9-modulated circRNA expression, we first evaluated whether DHX9 phosphorylation is induced by oxaliplatin treatment. The results of immunoprecipitated DHX9 from HCT116 treated with oxaliplatin showed a higher signal in the immunoblot using an antibody against the phospho-S/TQ motif (Figure 4C, left). A reciprocal immunoprecipitation using an anti-pS/TQ antibody followed by an immunoblot using an anti-DHX9 antibody had the same increasing pattern (Figure 4C, middle and right panels), implying that these two phosphorylation sites close to dsRBD may be regulated by oxaliplatin-induced molecular pathways. Accordingly, the pretreatment with PI3KK inhibitors decreased the induction of circCCDC66 (Figure 4D), suggesting that oxaliplatin-induced PI3KK activity may be involved in oxaliplatin-induced circCCDC66 expression. To further characterize the individual roles of S279 and S321 phosphorylation, HCT116 cells were transfected with wildtype DHX9, nonphosphorable mutant 279A, 321A or 279A/321A. Cells expressing the 279A mutant had a marginal effect on oxaliplatin-induced circCCDC66 expression (Figure 4E, left panel: no statistical difference between wildtype oxaliplatin (WT/oxa) or 279A/oxa), while cells expressing the 321A mutant diminished oxaliplatin-induced circCCDC66 expression (Figure 4E, left panel: WT/oxa vs. 321A/oxa). The combinational mutant did not further decrease the level of circCCDC66 (Figure 4E, left panel: AA bars). Coherent to the previous results of posttranscriptional regulation (Figure 3B, right), the levels of CCDC66 mRNA were not affected by these DHX9 mutants (Figure 4E, right). The differential effects of exogenous wildtype or mutated DHX9 were not caused by the different levels of exogenous DHX9 (Figure 4F). More importantly, the cells expressing nonphosphorable versions of DHX9 were less potent to develop resistant colonies (Figure 4G), suggesting that PI3KK-mediated DHX9 phosphorylation may be one of the oxaliplatin-modulated pathways for circRNA biogenesis.

## 3. Discussion

CircCCDC66 plays important roles during the development of chemoresistance in cancer. The oncogenic roles of circRNA have been widely demonstrated in various cancers. Our previous study reported that the expression of circCCDC66 is correlated to precancerous polyps and cancerous tissues and contributes to the development of colorectal cancer through the preferential protection of oncogenes [13]. The failure of treatments for colorectal cancers commonly results from the development of chemoresistant tumors accompanied with metastasis. In a previous study, the role of circCCDC66 during epithelial-mesenchymal transition (EMT), a process highly related to metastasis, was further extended in lung cancer [32]. The level of circCCDC66 was positively correlated to the mesenchymal transformation. The knockdown experiment implied that focal adhesion kinase (FAK) may be involved in the regulation of circCCDC66 expression during EMT. However, the molecular mechanism underlying FAK-regulated circCCDC66 expression during EMT remains elusive. The development of chemoresistance and metastasis of a tumor generally take place during the progression of CRC and ultimately cause the failure of the treatment. Supporting the role of increased circCCDC66 in CRC patients with worse prognoses [13], our current study demonstrated that the expression level of circCCDC66 is elevated in oxaliplatin-resistant cells and directly induced by the treatment with oxaliplatin (Figure 1C,D, Figure 3A,B). Further analyses by applying the previously published list of circCCDC66-associated miRNAs and circCCDC66 targets in the transcriptomic profiles from circCCDC66 knockdown cells [12,13] revealed the enrichment of predicted circCCDC66 targets in the gene list from the circCCDC66 knockdown experiment, implying that the gene/miRNA interaction might be feasible to predict the targets of circRNA. Thus, circCCDC66-inhibited apoptosis may be likely mediated through a multiple miRNAs-regulated gene network (Figure 2C,D). However, without oxaliplatin-induced cellular stress, circCCDC66 knockdown is not sufficient to induce apoptosis (Figure 3D,E). These results are coherent to our previous report that the loss of circCCDC66 mainly causes the retardation of proliferation, but not cell death, without the genotoxic stress [13]. Taken together, circCCDC66 exerts multifaceted roles during the development of CRC. In the early stage, it promotes cell proliferation, invasion and migration, while, in the advanced stage, the elevated expression of circCCDC66 modulated by EMT and/or chemotherapeutic stimulation may contribute to the development of chemoresistance and recurrence. Further investigations to finely dissect its role will reveal more insight and help the development of therapeutic strategies to cope with CRC.

**Figure 5:**
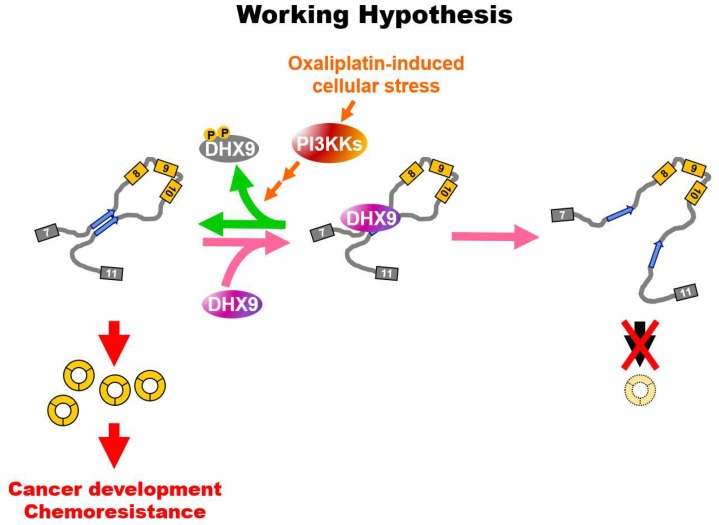
Phosphorylated DHX9 favors circRNA expression. The illustration of distinct populations of DHX9. Genotoxic stress-induced phosphorylated DHX9 may have a lower capacity to access RNA substrates, favor the interaction between intronic sequences and, thus, allow the occurrence of backsplicing to produce circRNAs.

DNA damage responses involve the modulation of multiple molecular events in the central dogma, such as the regulation of transcription, splicing and translation [26,33,34]. However, how it might regulate the biogenesis of the circular transcript remains largely unknown. In this study, we first provided evidence supporting that the expression of circular RNA is induced by oxaliplatin through PI3KK-mediated DHX9 phosphorylation (Figure 5). A pioneer study demonstrated that the decrease of DHX9 causes RNA to form secondary structures, which, in turn, promote the occurrence of backsplicing [17], indicating the critical role of DHX9 for circRNA production. DHX9 is upregulated in tumorous tissues and thought to be a potential therapeutic target due to its positive roles in the regulation of cell survival under treatments with chemotherapeutic compounds [20,22,35]. The upregulation of DHX9 can be part of the compensation for the increased loading of transcription and splicing machineries [36]. In our current report, we first found that the level of circCCDC66 was induced by the oxaliplatin treatment (Figure 3A,B), but the level of DHX9 was not affected (Figure 4C), unlike the previous model, in which the expression of circRNAs is induced by the decreased level of DHX9 [17]. Our proposed model is that phosphorylation near the substrate binding sites may hinder the capacity of DHX9 to resolve RNA pairing, providing a novel and alternative mechanism to explain the link between oncogenic circRNA and upregulated levels of DHX9 in tumors. DHX9 with multiple statuses regulated by posttranslational modifications may exert distinct functions in cancer cells and is worth further investigation.

DHX9 is involved in the multiple pathways of DNA damage responses. DHX9 was found to be associated with and phosphorylated by DNA-dependent protein kinase (DNA-PK) in an RNA-dependent manner [37], and yet, the role of PI3KK in the RNA metabolism or the identity of RNA in this context remains uncharacterized. The exact site of DNA damage response-induced phosphorylation on DHX9 was revealed in a later study which identified the sites of DNA damage-induced phosphorylation at S321 through a mass spectrum-based screening [30]. Compared to phosphorylation at S321, phosphorylation at S279 played a marginal role, since 279A did not alter the potential of oxaliplatin-induced circCCDC66 expression (Figure 4E), and phosphorylation at this site may not be related to genotoxic stress at all [38]. By combining the role of DHX9 on resolving RNA secondary structures and DNA damage-induced PI3KK-mediated phosphorylation, we proposed a possible mechanistic explanation for circRNA biogenesis, mirroring the model that DHX9 downregulation promotes circRNA expression [17]. The hypofunction of DHX9 was induced by oxaliplatin-triggered cellular stress through PI3KK-mediated phosphorylation near a double-strand RNA binding domain, allowing pairing between intronic sequences and, thus, promoting the expression of circRNAs. We speculated that negative charges contributed by phosphorylated serine residue(s) may disrupt the interaction between DHX9 and the RNA substrate. The phosphorylated DHX9 may harbor a suboptimal efficacy to process the pairing between the intronic sequences. Interestingly, DHX9 is also known to promote gene expression [20], but the phosphorylation of DHX9 is dispensable for its transactivation [29], implying that there may be different molecular pools of DHX9 with/without phosphorylation for either the regulation of splicing or transactivation. Our discovery provides an alternative point of view that targeting DHX9 with differential molecular statuses will be critical when developing a strategy against DHX9 to tackle cancer. The DHX9-suppressing tumor cells may gain advantages from the deficient RNA processing-induced oncogenic circRNAs and escape from the therapeutic treatment against DHX9. How to cope with DHX9 with differential phosphorylation statuses and/or the subsequent induction of oncogenic circRNA(s) will be a pivotal task and warrant further investigation in the near future.

## 4. Materials and Methods 

### 4.1. Cell Culture and Treatment

Cells used in this study (HCT116 and HT-29) originated from the American Type Culture Collection (ATCC, Manassas, VA, USA) and were cultured under the conditions according to ATCC recommendation, in a humidified atmosphere with 5% CO_2_ at 37 °C. Cells were maintained under 90% confluence and reseeded in desired cell numbers for corresponding containers. The oxaliplatin-resistant (OxR) cell lines were established by treating cells with an initial high dose of oxaliplatin (10 μg/mL) for the first week, followed by treatment with 1-μg/mL oxaliplatin for the subsequent 3 weeks. The resistant cells were maintained at 250-ng/mL oxaliplatin and were resumed to oxaliplatin-free medium 2 days prior to further experiments. For collecting apoptotic cells, the floating cells in medium and attached cells were both collected at indicated time points.

### 4.2. RNA Preparation, Reverse Transcription and Quantitative PCR

Cells were lysed in TRI reagent (Sigma-Aldrich, St. Louis, MO, USA), and total RNA was isolated according to the manufacturer’s instructions. The concentration of RNA was determined by UV absorption at 260 nm using a micro-volume spectrophotometer (Maestrogen MN-913A, Hsinchu City, Taiwan). Total RNA (1 μg) was proceeded to reverse transcription by using a random hexamer at 42 °C for 90 min, and one-tenth of the reverse transcription product then underwent SYBR green-based quantitative real-time PCR (normalized to 18S ribosomal RNA using the 2^−ΔΔCT^ method) using Applied Biosystems StepOnePlus™ Real-Time PCR System (Applied Biosystems, Foster City, CA, USA). The sequences of primers used in this study are listed in Supplementary Table S1.

### 4.3. Transfection

Small interfering RNA (siRNA) oligonucleotides used to target circCCDC66 were adapted from the previous study [13] and are also listed in Supplementary Table S2. Cells were transfected with siRNA oligonucleotides against circCCDC66 or oligonucleotides for negative control (siCON; Dharmacon, D-001810-10-20) at a final concentration of 50 nM using Lipofectamine 2000 (Life Technologies, Carlsbad, CA, USA) reagent according to the manufacturer’s instructions. The construct for the exogenous expression of DHX9 was made by adding C-terminal FLAG tag to pUNO1-DHX9 (InvivoGen, San Diego, CA, USA), followed by the replacement of a blastidin resistance cassette with ampicillin one from pIS2 (the resultant backbone was denoted as pUNO1A) [39,40]. The S279A and S321A mutants were generated by using site-directed mutagenesis. Primers sequences are listed in Supplementary Table S2.

### 4.4. Cell Survival and Caspase 3 Activity Assays

Cells were seeded in the 12-well plate (2 × 10^5^/well) and treated with oxaliplatin (0.1, 1 and 10 μg/mL) on the next day. After incubation for 48 h, the cells were fixed and stained in Coomassie blue solution (0.05% Coomassie brilliant blue R-250, 50% methanol, 10% acetic acid). For cells transfected with siRNA targeting circCCDC66, the cells were reseeded to 12-well plate 24 h post-transfection and treated with oxaliplatin for another 48 h. The caspase 3 activities were determined by spectro-photometric detection of released *p*-nitroaniline (pNA) from the labeled substrate DEVD-pNA according to the manufacturer’s instructions (BioVision, Cat#: K106, Milpitas, CA, USA).

### 4.5. Clonogenic Assay

To assay the development of chemoresistance in CRC cell lines, cells were seeded in 6-cm dish (2 × 10^4^ cells) and treated with 1-µg/mL oxaliplatin for more than 7 days. For siRNA knockdown, the cells were transfected with siRNA for 6 h and reseeded for treatment with 1-µg/mL oxaliplatin. For cells expressing exogenous DHX9, the cells were transfected with DHX9-expressing constructs (pUNO1A-DHX9-WT, S279A or S321A) for 16–18 h and then reseeded for treatment with 1-µg/mL oxaliplatin.

### 4.6. Immunoprecipitation and Immunoblot

Total proteins (20–30 μg) were resolved and immunoblotted as routinely performed in our laboratory by using the following antisera: anti-cleaved caspase 3 (Cell Signaling Technology, Cat#: 9661, Danvers, MA, USA), anti-β-actin (Sigma-Aldrich, Cat#: A5441, St. Louis, Missouri, USA), anti-DHX9 (Abcam, Cat#: ab183731, Cambridge, UK), anti-FLAG (Sigma-Aldrich, Cat#: F3165) and anti-vinculin (Millipore Corp., Cat#: MAB3574, Burlington, MA, USA). For assessing DHX9 phosphorylation, the immunoprecipitated DHX9 by using anti-DHX9 antibody (Abcam, Cat#: ab183731, Cambridge, UK) was probed with anti-phospho-(S/T)Q antibody (Cell Signaling Technology, Cat#: 2851, Danvers, MA, USA). In a reciprocal way, the immunoprecipitated phospho-PI3KK substrates (harboring the phospho-Ser/Thr-Gln motif) were probed with antibody against DHX9. Briefly, cell lysates prepared in NTEN buffer (150-mM NaCl, 25-mM TrisCl, pH 7.5, 1-mM EDTA and 1% NP-40) were precleared with 20 μL of Dynabeads^®^ Protein G (Invitrogen, Cat#: 10003D, Carlsbad, CA, USA) at room temperature for 1 h, followed by overnight incubation with the antibody for immunoprecipitation at 4 °C on a rotary shaker. The beads/antibody complexes were washed with NTEN buffer for 3 times and then eluted and boiled in 2× sample buffer (20-mM TrisCl, pH7.5, 20-mM EDTA, 2% SDS, 20% glycerol, 0.004% bromophenol blue and 200-mM dithiothreitol). The immunoprecipitated proteins were then resolved on the SDS-PAGE and probed with the antibody against phospho-(S/T)Q or DHX9 to assess the level of phospho-DHX9. All original figures of western blot can be found in the Supplementary File.

### 4.7. RNA Sequencing and Bioinformatic Analyses

Total RNA from oxaliplatin-resistant HCT116 cells transfected with siRNA control oligonucleotides or ones against the backsplice junction of circCCDC66 for 24 h was isolated using TRI reagent (Sigma-Aldrich, St. Louis, MO, USA) according to the manufacturer’s instructions and quantified by using Agilent 2100 Bioanalyzer (Agilent Technologies, Santa Clara, CA, USA). The ribosomal RNA was depleted by using Ribo-zero rRNA Removal Kit (Epicentre, Madison, WI, USA), and the subsequent library was constructed by using NEBNext Ultra Directional RNA Library Prep Kit for Illumina (New England Biolabs, Ipswich, MA, USA). The RNA-seq was performed on the Novaseq 6000 platform (paired-end reads, 2 × 150 bp), and the data were deposited to the Sequence Read Archive hosted by the National Center for Biotechnology Information (Bethesda, MD, USA) under the accession number of BioProject: PRJNA607108. Raw reads were processed to remove low-quality reads, adaptors and rRNA sequences by using FASTQ preprocessor and BBMap [41,42], followed by alignment to the human reference genome (GRCh38.92) using the BWA-MEM algorithm (bwa-0.7.17) [43]. Identification of the circular RNAs was implemented by using CIRI2 (v2.0.6) and in-house scripts [44]. Expression levels of individual genes were analyzed by using HTSeq (v0.11.2) and edgeR (v3.24.3) [45,46]. The reads among samples were normalized to the number of total valid reads. The predicted circCCDC66 target genes [12,13] were then used as a custom gene set and evaluated in Gene Set Enrichment Analysis (GSEA) [47] by using the sorted ratios of reads between treatments from RNA-seq. Pathway enrichment analysis was performed at ICGC data portal [48].

### 4.8. Statistical Analysis

All data were expressed as mean ± standard error of the mean (SEM). Student’s *t*-test was performed for comparison between two groups if the data fit the normal distribution; otherwise, the Mann-Whitney test was applied instead. For three or more groups, differences among groups were determined by one-way ANOVA followed by Tukey’s multiple test using GraphPad Prism 5.0 (GraphPad Software, Inc. La Jolla, CA, USA). Statistical significance was set at *p* < 0.05.

## 5. Conclusions

In this study, we discovered that the oxaliplatin-resistant CRC cells express higher levels of oncogenic circRNA CCDC66, and the expression of circCCDC66 is induced by oxaliplatin-induced cellular stress through DHX9 phosphorylation in CRC. The induction of circCCDC66 is dependent on the treatment with oxaliplatin and is required for the establishment of chemoresistance to oxaliplatin. The CRC cells with circCCDC66 knockdown showed severer apoptosis and downregulation of several genes related to the modulation of the cell cycle and apoptosis. Moreover, the induction of circCCDC66 is caused by the hypofunction of DHX9, which is triggered by oxaliplatin-induced PI3KK-mediated phosphorylation near the substrate-binding domains. Blockage of PI3KK activity or hindrance of DHX9 phosphorylation by point mutation attenuated the oxaliplatin-induced circCCDC66 expression and the establishment of chemoresistant cells. Taken together, this study provides an insightful mechanism for how CRC cells may escape from chemotherapeutic treatment through impaired RNA processing-induced oncogenic circRNA.

## Supplementary Materials

The following are available online at https://www.mdpi.com/2072-6694/12/3/697/s1: Table S1: Primer sequences. Table S2: Sequences of siRNA oligonucleotides. Supplementary file: All original figures of western blot.
