# Supplementary material for: Oxaliplatin-Induced DHX9 Phosphorylation Promotes Oncogenic Circular RNA CCDC66 Expression and Development of Chemoresistance
Source: Cancers (Basel). 2020 Mar 16;12(3):697. doi: 10.3390/cancers12030697 (PMC7140115; doi:10.3390/cancers12030697)
Supplement: Supplementary file 1 [file cancers-12-00697-s001.zip › cancers-723559-suppl-final/cancers-723559 -suppl-final.docx]

Article

Oxaliplatin-Induced DHX9 Phosphorylation Promotes Oncogenic Circular RNA CCDC66 Expression and Development of Chemoresistance

Ya-Chi Lin, Ya-Shan Yu, Hui-Hsuan Lin and Kuei-Yang Hsiao

Supplementary Materials

**Supplementary Table S1.** Primer sequences.

**Primer ID	Sequence (5' to 3')	Notes**

CCDC66 e10 F	TCTCTTGGACCCAGCTCAG	qPCR for circular transcript

CCDC66 e9 R	TGAATCAAAGTGCATTGCCC	(spanning the junction)

CCDC66 e16 F	ACCTTGATCCCGATGCACC	qPCR for linear transcript

CCDC66 e17 R	GATTGCTTGCTGTCGATCCC	of CCDC66

18S rRNA F	GTGTGCCTACCCTACG	qPCR

18S rRNA R	TGACCCGCACTTACTG

AmpR NdeI F	GTCATATGATCGATAAGGATCCAGGTGG	for subcloning bla-AmpR

AmpR EcoRI R	TATGAATTCAAACTTGGTCTGACAGTTACC	from pIS2 to pUNO1-DHX9

DHX9_cFLAG F	ACAAGGATGACGATGACAAGTAAGC

TAGCTGGCCAGACATGAT	add FLAG to C-terminus of

DHX9_cFLAG R	TCATCGTCATCCTTGTAGTCATAGC	DHX9

CGCCACCTCCTCTTC

DHX9 S279A F	CCTTACAAAGTAAACCTCGCTCAAGATTTAGAGCATC	mutates Ser279 to Ala

DHX9 S279A R	GATGCTCTAAATCTTGAGCGAGGTTTACTTTGTAAGG

DHX9 S321A F	CTCAGTTCGAACCAGCTCAGCGACAAAACC	mutates Ser321 to Ala

DHX9 S321A R	GGTTTTGTCGCTGAGCTGGTTCGAACTGAG

**Supplementary Table S2.** Sequences of siRNA oligonucleotides.

**Name	Target Sequence (5' to 3')	Manufacturer/Cat. #	Notes**

siCON	UGGUUUACAUGUCGACUAA	Dharmacon/D-001810-10-20	negative control

UGGUUUACAUGUUGUGUGA

UGGUUUACAUGUUUUCUGA

UGGUUUACAUGUUUUCCUA

siCCDC66	CAAUUAGAGCAUCAGGAAA	Dharmacon/Custom synthesis	target CCDC66 exon 10/8

GAGCAUCAGGAAACAGUAC	Dharmacon/Custom synthesis	backsplice junction

|  | © 2020 by the authors. Licensee MDPI, Basel, Switzerland. This article is an open access article distributed under the terms and conditions of the Creative Commons Attribution (CC BY) license (http://creativecommons.org/licenses/by/4.0/). |
| --- | --- |
